# The anti-inflammatory and anti-apoptotic effects of gallic acid against mucosal inflammation- and erosions-induced by gastric ischemia-reperfusion in rats

**Published:** 2015-12-15

**Authors:** Seyyed Ali Mard, Shahnaz Mojadami, Yaghoob Farbood, Mohammad Kazem Gharib Naseri

**Affiliations:** 1 *Physiology Research Center (PRC), Research Center for Infectious Diseases of Digestive System, Department of Physiology, School of Medicine, Ahvaz Jundishapur University of Medical Sciences, Ahvaz, Iran; *; 2 *Physiology Research Center (PRC), Department of Physiology, School of Medicine, Ahvaz Jundishapur University of Medical Sciences, Ahvaz, Iran.*

**Keywords:** Caspase-3, Gallic acid, Inducible nitric oxide synthase, Ischemia-reperfusion injury, Rat

## Abstract

The present study aimed to evaluate the protective effect of gallic acid on gastric mucosal lesions caused by ischemia-reperfusion (I/R) injury in rat. Forty male rats were randomly divided into sham, control (I/R injury) and three gallic acid-pretreated groups. To induce I/R lesions, the celiac artery was clamped for 30 min and then the clamp was removed to allow reperfusion for 6 hr. Pretreated rats received gallic acid (15, 30 or 60 mg kg^-1^, intraperitoneally) 30 min prior to the induction of I/R injury. Macroscopic and microscopic evaluations of the areas of ulceration were compared. Samples of gastric mucosa were collected to evaluate the protein expression of pro-apoptotic factor, caspase-3, and pro-inflammatory enzyme, inducible nitric oxide synthase (iNOS) using western blot. Pretreatment with gallic acid decreased the total area of gastric lesions. Gallic acid at 30 mg kg^-1^ decreased the levels of protein expression of caspase-3 and iNOS induced by I/R injury. Our findings showed the protective effect of gallic acid on gastric mucosa against ischemia-reperfusion injury. This effect of gallic acid was mainly mediated by reducing protein expression of iNOS and caspase-3.

## Introduction

It is well established that reactive oxygen species (ROS) and reactive nitrite species (RNS) are involved in development of gastric ischemia-reperfusion injury.^1^^,^^2^ Also, contribution of nitric oxide (NO) in I/R-induced gastric lesions has been documented.^3^ It has been shown that NO reacts with ROS and produces highly toxic substances such as peroxynitrite and singlet oxygen.^4^^,^^5^ Naturally, NO is produced from L-arginine in mammalian tissues by three isoforms of NO synthase, two constitutive (eNOS and nNOS) and one inducible (iNOS) form. In the oxidative stress iNOS gene transcription and promoter activity are increased and it regulates chromatin modification leading to cellular injury.^6^^,^^7^

Acute gastric mucosal injury has been reported as a result of decreased blood flow and alterations in gastric microvascularization,^8^ that leads to ischemia reperfusion injury (I/R). The ischemia followed by reperfusion during gastric I/R injury develops mucosal inflammation which in turn often leads to hemorrhagic types of inflammation. These changes serve as a perquisite for mucosal erosions.^9^ The pathophysiology of I/R-induced injury is associated with inflammatory responses that elicit tissue damage. The acute inflammatory response is characterized by induction of inflammatory cytokines,^10^ neutrophil infiltration,^11^ and generation of oxygen free radicals.^1^^,^^12^

Gallic acid is also known as 3,4,5-trihydroxybenzoic acid, found in gallnuts, sumac, witch hazel, green tea, oak bark, pineapple, grapes and other plants.^13^ Gallic acid has been shown to possess strong anti-oxidative activity and many reports have shown that neuro-, cardio-, and hepato-protective effects of gallic acid.^14^^-^^17^ Additionally, gallic acid has been shown to protect the gastric mucosa against ethanol-, and aspirin-induced gastric lesions in rats.^18^^,^^19^ The gastric I/R-injury is an important and common clinical problem which could result in mucosal erosions and ulcers. There are some clinical conditions that are contributed to gastric I/R injury-including peptic ulcer bleeding, vascular rupture or surgery, ischemia gastrointestinal disease and hemorrhagic shock.^20^ However, the satisfactory clinical methods to treat the gastric I/R injury are rare.^21^^,^^22^ Therefore, promoting the tissue levels of antioxidants by pretreatment therapy can attenuate tissue damages following ischemia-reperfusion-induced injury. To the best of our knowledge, there is no report about the effect of gallic acid on gastric mucosa following I/R injury in rat. Therefore, the aim of the present study was to evaluate the protective effect of gallic acid on gastric mucosal lesions induced by I/R injury in rat by evaluating the changes in the level of the protein expression of apoptotic factor, caspase-3, and pro-inflammatory protein, inducible nitric oxide synthase (iNOS) in gastric mucosa. 

## Materials and methods

**Animals.** Forty male Wistar rats (body weight 150 to 200 g) were purchased from the animal house of Ahvaz Jundishapur University of Medical Sciences, Ahvaz, Iran. The animals were fed on conventional diet and had free access to tap water. They were maintained under standard conditions of humidity, temperature (22 ± 2 ˚C) and 12 hr light/dark cycle. The animals were deprived of food but not water overnight before intervention. All experiments were carried out in accordance with the regulations set by ethics committee of Ahvaz Jundishapur University of Medical Sciences (research project PRC156). 

**Chemicals. **Gallic acid (G7384; Purity: 100 ± 2.5%), was purchased from Sigma (St. Louis, USA). Ketamine hydrochloride and xylazine hydrochloride were purchased from Alfasan (Woerden, The Netherlands).

**Animal grouping and experimental procedures.** The rats were randomly assigned to equal five groups (n = 8): Control, gallic acid-treated (15, 30 or 60 mg kg^-1^, intraperitoneally) and sham groups. Gastric I/R injury was induced according to the method of Wada *et al*.^23^ Briefly, under an intraperitoneal injection of 60 mg kg^-1^ ketamine and 15 mg kg^-1^ xylazine mixture), the rats underwent a midline laparotomy and the celiac artery was carefully isolated from its adjacent tissues. The celiac artery was then clamped for 30 min to induce ischemia and the clamp was removed to allow reperfusion for 6 hr.

Depth of anesthesia was checked by pedal withdrawal (toe pinch) reflex every 15 min during ischemic episode and immediately after removal of the clamp, the animals were returned to individual fasted cage and allowed to recover from anesthesia. If the pedal withdrawal reflex was observed during the ischemic period, a supplemental dose of ketamine and xylazine (one third of the initial dose) was administered to maintain adequate anesthesia. At the end of experiment and end of reperfusion period animals were anesthetized again and their stomach was removed. Animal body temperature was measured with a rectal thermometer and maintained at 37 ˚C using a homeothermic blanket control system (Harvard, UK). Sham-operated rats underwent laparotomy without inducing I/R injury. To investigate the gastroprotective effect of gallic acid against mucosal damage induced by I/R injury, 3 groups of animals received intraperitoneal gallic acid at doses of 15, 30 or 60 mg kg^-1^, 30 min prior to I/R injury. At the end of experiment, animals were euthanized by cardiac exsanguination. In order to calculate the gastric mucosal lesions, the stomachs of animals were removed, opened along the greater curvature, rinsed with physiological saline and pinned out in ice-cold saline. To calculate the degree of gastric lesions, the total area of mucosal lesions were measured by Image J software. The lesion area is expressed as the percentage of the total area of the glandular stomach except for the fundus using the following formula:^24^

$\mathrm{UI}\left(\%\right)=\frac{\mathrm{Ulcerated area}}{\mathrm{Total stomach area expect fundus}}\times \mathrm{100}$

where, UI stands for “ulcer index”. 

Immediately after taking photo of the stomachs for measurement of the surface area of gastric lesions, samples of gastric mucosal tissue (100 mg, in each) including the lesion’s area and the surrounding ulcer margin up to 5 mm were quickly excised, snap-frozen and stored in liquid nitrogen for molecular analysis. The macroscopic evaluation of gastric mucosal lesions showed that the optimal protective dose of gallic acid against I/R injury was 30 mg kg^-1^. Therefore, the molecular analysis was carried out in animals that received the optimal dose. 

**Evaluation of microscopic mucosal damage. **For histological evaluation, stomachs from all groups were fixed in 10% formalin, dehydrated in grade ethanol, and embedded in paraffin. Thereafter, 5 µm sections of tissue were cut, stained with hematoxylin and eosin and assessed microscopically (Model IX50; Olympus Optical Co. Ltd., Tokyo, Japan) under 100× magnification.

**Protein extraction. **The tripure isolation reagent (Roche diagnostics, Indianapolis, USA) was used to extract the total proteins from gastric mucosal tissue, according to the manufacturer instructions. To analyze the protein fraction, protein pellets obtained using the tripure isolation reagent from gastric mucosal samples, were resuspended in 1% sodium dodecyl sulfate (SDS). The total recovery and integrity of these fractions were determined by Bradford assay and SDS–polyacrilamide gel electrophoresis. 

**Western blot analysis. **Mucosal proteins were separated by SDS-PAGE on 10% acrylamide gels and were transferred onto a nitrocellulose membrane. The membranes were blocked with 5% non-fat dry milk dissolved in tris-buffered saline with 0.1% tris-buffered saline, 0.1% tween 20 (TBST; pH 7.6) for 6 hr and then incubated overnight at 4 ˚C with 1:400 dilution of rabbit polyclonal anti-iNOS antibody (ab95441; Abcam, San Francisco, USA), 1:1000 dilution of rabbit polyclonal anti-Caspase-3 antibody (ab90437; Abcam) or dilution 1:5000 of mouse monoclonal anti-beta actin antibody (ab20272; Abcam) was added to the membrane. After five washes with TBST, membranes were incubated with a 1:7000 dilution of rabbit polyclonal secondary antibody to mouse IgG HRP; for 2 hr at room temperature. Labelled proteins were detected using a chemiluminescence western blotting system. The expression of studied proteins was semi-quantified by Image J analysis software and the values were normalized to β-actin.

**Statistical analysis. **Data are shown as mean ± SEM. Statistical analysis was performed by one-way ANOVA and followed by post-hoc Turkey's test in SPSS (version 16; SPSS Inc., Chicago, USA). Significance was set at a *p* < 0.05 level. 

## Results

**Fig. 1:**
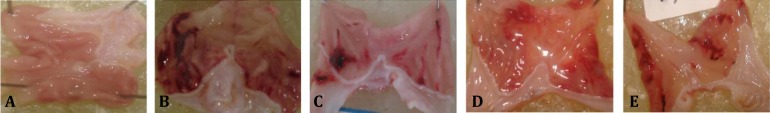
Gross appearance of the stomachs. Sham-operated group (A): Normal appearance of gastric mucosa; I/R injury control (B): Severe mucosal lesions 6.5 hr after inducing gastric ischemia-reperfusion injury. GA15 (C), GA30 (D), and GA60 (E): Gallic acid pretreatment (15, 30 and 60 mg kg^-1^, respectively) decreased the mucosal lesions induced by ischemia-reperfusion

**Fig. 2:**
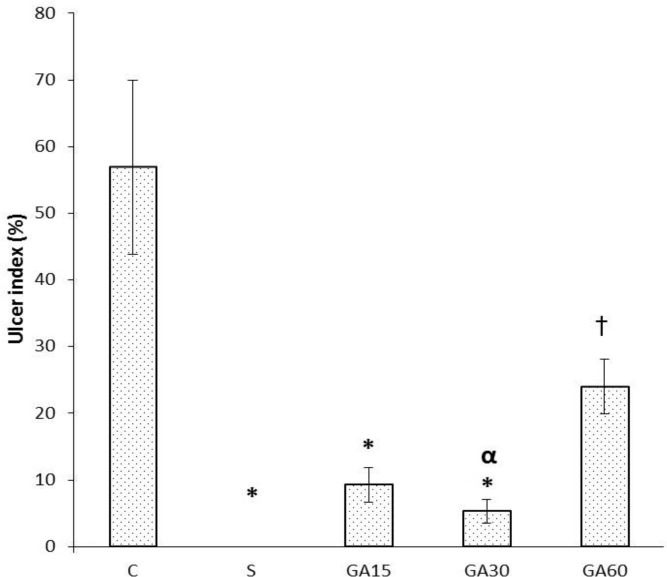
The calculated ulcer index in C: I/R injury control animals, S: Sham-operated group, GA15, GA30, GA60: Rats pretreated with gallic acid (15, 30 and 60 mg kg^-1^, intraperitoneal) 30 min before intervention

**Fig. 3:**
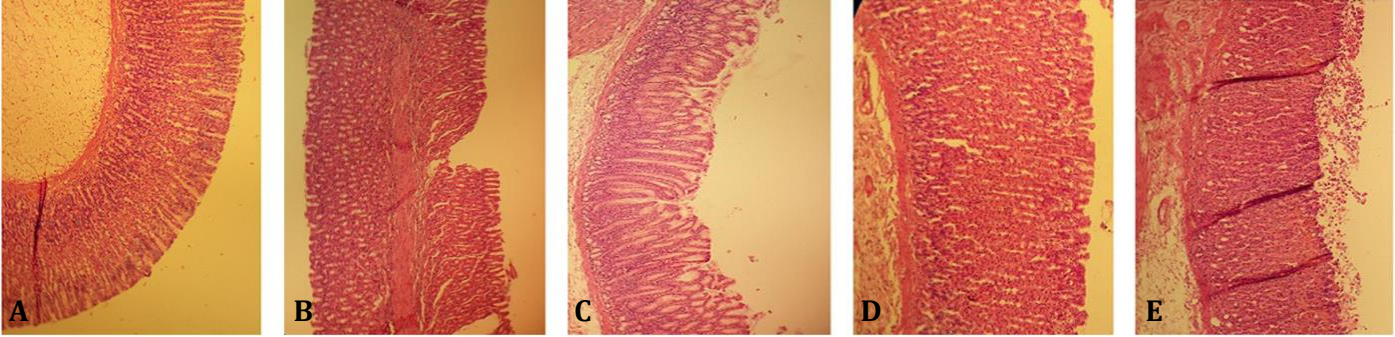
Histological evaluation of the gastric mucosa. Representative gastric sections were obtained 6.5 hr after I/R injury. Sham-operated group: Depicts no disruption to the surface epithelium, I/R control Shows multiple erosions, exfoliation and necrosis of superficial cells, hemorrhages in the mucosal layer and severe alterations in the architecture of glandular parts of the gastric mucosa; GA: Rats pretreated with gallic acid (15, 30 and 60 mg kg^-1 ^30 min before intervention), demonstrate moderate disruption of the surface epithelium (Hematoxylin and eosin, 100×).

**Effect of gallic acid pretreatment on gastric mucosal lesions induced by I/R injury. **Macroscopic evaluation of the stomachs showed that there was no gastric mucosal lesion in sham-operated animals. Gallic acid pretreatment decreased gastric mucosal lesions induced by ischemia-reperfusion injury (Fig. 1). As shown in Figure 2, pretreatment with gallic acid decreased the gastric lesions induced by I/R injury. The total areas of gastric lesions in gallic acid-pretreated rats were significantly lower than in control I/R injury animals. The ulcer index in I/R injury control rats was 56.89 ± 13.10 which was significantly higher than in 15, 30, and 60 mg kg^-1 ^pretreated gallic acid-treated groups (9.3 ± 2.57, *p *< 0.001; 5.34 ± 1.80, *p *< 0.001; and 24.02 ± 4.10, *p* < 0.01; respectively), (Fig. 2). Pretreatment with gallic acid (15, 30 and 60 mg kg^-1^, Figs. 1 and 2) attenuated the gastric lesions induced by I/R injury. The inhibition percent of acute gastric ulcer lesions against I/R injury in gallic acid-pretreated groups (15, 30 and 60 mg kg^-1^) were 85.73, 90.62, and 51.88, respectively. The optimal dose of gallic acid found to be 30 mg kg^-1^. Microscopic examinations showed gastric lesions with multiple erosions, exfoliation and necrosis of superficial cells, hemorrhages in the mucosal layer, marginal inflammation and neutrophil aggregation and severe alterations in the architecture of glandular parts of the gastric mucosa 6 hr after ischemia (Fig. 3). No gastric damage was observed in the gastric mucosa of sham-operated rats (Fig. 3).

**Fig. 4:**
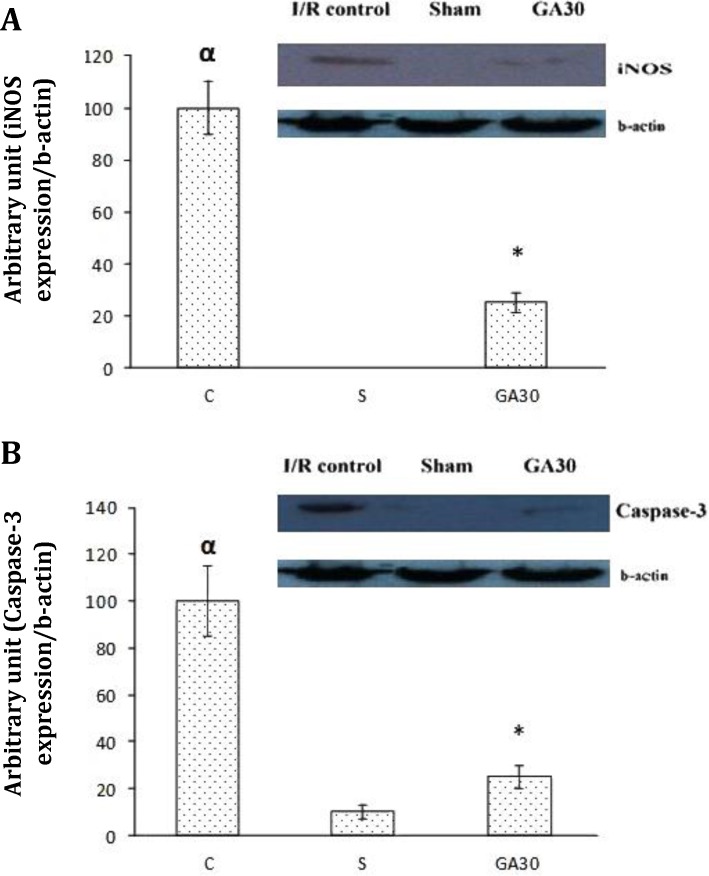
Western blot analysis for protein synthesis of iNOS (A) and caspase-3 (B) in the rat gastric mucosa following, C: I/R injury control, GA15: Gallic acid (15 mg kg^-1^, intraperitoneal), S: Sham-operated group. Gallic acid pretreatment significantly decreased protein expression of iNOS and caspase-3. Data are expressed as mean ± SEM. The protein levels were normalized to β-actin

**Effect of gallic acid pretreatment on mucosal protein expressions of caspase-3 and iNOS. **As shown in Figure 4, the level of protein expression of iNOS in I/R injury control rats was significantly higher than in gallic acid (30 mg kg^-1^) pretreated animals (*p* < 0.01). Figure 4, also shows that inducible nitric oxide synthase (iNOS) was not expressed in sham-operated group. Thirty min gastric ischemia followed by 6 hr reperfusion significantly increased the basal level of caspase-3 expression compared to the sham-operated animals (*p* < 0.01). This level was significantly decreased by gallic acid pre-treatment (*p *< 0.01).

## Discussion

The results of this study showed that: (1) pretreatment with gallic acid decreased the total surface area of the acute gastric mucosal lesions induced by I/R; (2) the level of protein expression of iNOS was lower in rats pretreated with gallic acid at 30 mg kg^-1^ than in the control I/R injury rats and (3) the basal level of protein expression of caspase-3 was higher in the control I/R injury rats than in rats pretreated with gallic acid at 30 mg kg^-1^ and sham-operated group.

Many studies have demonstrated the anti-oxidant and anti-inflammatory activities of gallic acid in different models of tissue injury. It has been reported that administration of ethanolic extact of *Ficus racemosa* Linn. stem bark, a major source of gallic acid, improves kidney function in mice. The renoprotective effect of gallic acid in this model may rely on its anti-oxidant activity by restoring serum biomarkers and tissue antioxidant status to near normal levels.^25^ Also gallic acid has been shown to protect kidney function against renal ischemia-reperfusion injury in rats by activating peroxisome proliferators activating receptor gamma (PPAR-γ).^26^ Gallic acid has also been shown to exert a hepatoprotective activity on paracetamol-induced liver damage in mice.^17^ The hepatoprotective mechanism of gallic acid is mediated through inhibition of lipid peroxidation, reducing the release of Tumor necrosis factors-alpha (TNF-α) and reversing the anti-oxidant status. Moreover, gallic acid extracted from Daylily flowers has been shown to increase the activity of superoxide dismutase (SOD) and to reduce the lipid peroxidation in both blood and liver of rat.^27^ In a similar manner, gallic acid and its novel synthetic derivative decrease the gastric mucosal lesions caused by aspirin plus pyrolus ligation, and ethanol-induced gastric ulcer by reducing the acid output, increasing the levels of anti-oxidant (SOD, catalase, and glutathione peroxidase), decreasing myeloperoxidase and lipid peroxidation, reducing the release of TNF-α, promoting endogenous defensive factor prostaglandin E_2_.^18^^,^^19^ We found that the level of protein expression of the pro-inflammatory mediator iNOS significantly was decreased in rats pretreated with gallic acid at 30 mg kg^-1^ compared with control I/R injury rats (Fig. 4). Accordingly, it has been shown that the administration of gallic acid has been reported to suppress b-amyloid neurotoxicity by down-regulating both mRNA and protein levels of inflammatory cytokines including iNOS, cyclooxygenase-2, interleukin-β and TNF-α.^28^ Gallic acid was also found to inhibit protein expression of iNOS and NO production in lipo-polysaccharide-activated macrophages.^29^ Taken together, these findings showed that the possible gastro-protective mechanism of gallic acid could largely be mediated by down-regulating of inflammatory cytokine, iNOS.

In order to gain insight into other possible mechanisms that mediate the beneficial effects of gallic acid against I/R injury, the molecular marker for apoptosis (expression of caspase-3) was also investigated. The results showed that gallic acid significantly decreased the protein expression of caspase-3. In a similar manner, it has been shown that the increased activity of caspase-9 in HeLa cells, a model for human epithelial cells, is resulted from H_2_O_2_-induced oxidative stress and apoptotic cell death is decreased by gallic acid pretreatment.^30^ Gallic acid has also been indicated to protect rat insulinoma clone m5F (RINm5F) β-cells against glucolipotoxicity.^31^ The mechanism of this protection was proposed to be mediated by decrease in caspase-3 activity, up-regulating mRNA expression of anti-apoptotic protein (Bcl-2) and down regulating protein expression of pro-apoptotic protein (i.e. caspase-3). Our *in vivo* findings are consistent with previous *in vitro* reports which suggested that the reduction in protein expression of pro-apoptotic protein was possible mechanism for the anti-apoptotic activity of gallic acid.^30^^,^^31^ Therefore, it could be concluded that the gastroprotective effect of gallic acid on I/R injury is partly mediated by decreasing the protein expression of caspase-3.

Our macroscopic finding also showed the optimal gastro-protective dose of gallic acid was 30 mg kg^-1^. Consistent with this result, it has been shown that the most potent neuroprotective effect of gallic acid against beta-amyloid neurotoxicity was 30 mg kg^-1^.^28^ Our macroscopic and microscopic findings showed the inhibition percent of acute gastric lesions following I/R injury in the highest studied dose of gallic acid (60 mg kg^-1^) that was significantly lower than dose of 30 mg kg^-1^. Therefore, gallic acid at 60 mg kg^-1^ was less effective than its optimal concentration of 30 mg kg^-1^, suggesting that gallic acid at 60 mg kg^-1^ could be pro-oxidant.^32^ Some previous literatures have shown that gallic acid acts as a pro-oxidant under certain condition such as high concentration. It has been reported that tannins, gallic acid, ellagic acid and tannic acid, at lower doses, 1 and 5 μM, protect the digestive cells of mussels (*Unio tumidus*) against H_2_O_2_-mediated DNA damage but at higher dose (10 µM) induce DNA damage (single strand breaks) in these cells. Therefore, the anti-oxidative properties of tannins may change to pro-oxidative activities at the higher concentration.^33^ To find the protective mechanism(s) of gallic acid, we only evaluated molecular mechanism of the effective dose. It seems that this was the limitation of the present study. 

In conclusion, the present study for the first time showed the gastroprotective effect of gallic acid on I/R injury. The findings of this study demonstrated that: Pre-treatment with gallic acid decreased the total area of acute gastric mucosal lesions induced by I/R- and the protein expression of pro-apoptotic protein, caspase-3, and pro-inflammatory enzyme, iNOS, in 30 mg kg^-1^ gallic acid-pretreated rats were lower than in the I/R injury control. 
